# “To get the baby out off the hook”: a prospective, longitudinal, multicenter, observational study about decision making in vacuum-assisted operative vaginal delivery

**DOI:** 10.1186/s12884-022-04440-5

**Published:** 2022-02-16

**Authors:** Alessandro Svelato, Alis Carabaneanu, Claudia Sergiampietri, Paolo Mannella, Sara D’Avino, Caterina De Luca, Martina Bartolone, Roberto Angioli, Antonio Ragusa

**Affiliations:** 1 Department of Obstetrics and Gynecology, San Giovanni Calibita Fatebenefratelli Hospital, Isola Tiberina, Via di Ponte Quattro capi, 39, 00186 Rome, Italy; 2 Department of Obstetrics and Gynecology, Prato General Hospital, Prato, Italy; 3 Department of Obstetrics and Gynecology, Apuane Hospital, Massa, Italy; 4 Department of Reproductive Medicine and Child Development, University of Pisa, Pisa, Italy; 5 Department of Maternal and Child Health and Urological Sciences, “Sapienza” University of Rome, Rome, Italy; 6 Department of Gynecology, University Campus Biomedico, Rome, Italy

**Keywords:** Heuristic, Problem Solving, Vacuum, Assisted Vaginal Birth, Operative vaginal delivery, Decision making

## Abstract

**Background:**

Since operative vaginal delivery may be risky for women and might cause neonatal complications, the aim of this study is to assess appropriateness of the procedure.

This is a prospective, longitudinal, multicenter, observational study and it was conducted in three Italian Obstetric Units (Pisa, Massa Carrara and Prato). All term pregnant women, either nulliparous and multiparous, with singleton pregnancy and a cephalic fetus, with spontaneous or induced labour, requiring vacuum-assisted delivery were enrolled.

Indications to operative vaginal delivery were grouped as alterations of fetal cardiotocography (CTG) patterns, delay/arrest of second stage of labour or elective shortening of second stage of labour. A board consisting of five among authors evaluated appropriateness of the procedure.

**Results:**

Overall, 466 women undergoing operative vaginal deliveries were included. Cardiotocography, classified as ACOG category 2 or 3 was the indication for vacuum assisted delivery in 253 patients (54.29%). Among these, 66 women (26.1%) had an operative vaginal delivery which was then considered to be inappropriate, while in 114 cases (45.1%) CTG traces resulted to be unreadable.

**Conclusion:**

Decision making process, which leads clinicians to go for operative vaginal delivery, is often influenced by shortness of time and complexity of the situation. Therefore, clinicians tend to intervene performing vacuum delivery without adopting critical analysis and without adequately considering the clinical situation.

**Tweetable abstract:**

Operative vaginal delivery might be a risky procedure and should be performed only when clinically indicated and after adequate critical analysis.

## Introduction

Although vacuum-assisted vaginal delivery is now considered a routine procedure and related complications are relatively uncommon, it might be a high-risk procedure both for women and newborns.

Every medical procedure may have benefits, risks and possible side effects and should respond to the Hippocratic aphorism “*Primum non nocere” (“First, do no harm”)*. Theoretically, a procedure is considered to be appropriate if expected benefits overcome expected costs [1–3]. Decision making process is based both on scientific evidence and on emotional capabilities.

According to Croskerry, errors in healthcare can be divided into three categories: no-fault errors, due to external factors outside the clinician control, system-related errors, caused by technical or organizational barriers, and lastly cognitive errors or biases, due to the clinician’s poor clinical reasoning [4]. The latter sometimes may lead to irrational procedures application.

In difficult or emotionally charged situations, such as emergencies, clinicians work under pressure. In these cases, their clinical decisions may be led by emotions, rather than by a rational process [5]. A typical example is the decision of carrying out operative vaginal delivery. Vacuum-assisted vaginal delivery rates are different in every country and range between 10 and 15% in the US, 10% and 13% in the UK [6, 7]. In Italy, this rate is lower, with a wide distribution from one region to another and even from one birth centre to a close one. Italian *National* Institute of *Statistics* (*Istat*) reported in 2013 a rate of 4,8% operative deliveries [8].

The aim of this study is to analyze appropriateness of indications for operative vaginal deliveries.

## Materials and methods

We conducted a prospective, longitudinal, multicenter, observational study in three Italian Obstetric Units (Pisa, Massa Carrara and Prato). These are three urban community hospitals (about 2000 deliveries/year each), where medical care is provided by attending obstetricians working in the National Health Service as most Centers in our country.

All term pregnant women (after 37 weeks gestation), nulliparous and multiparous, with singleton pregnancy and a cephalic fetus, either with spontaneous or induced labour, requiring vacuum-assisted delivery, who delivered between April 2017 and January 2019 were enrolled.

Exclusion criteria were: multiple pregnancy, non-cephalic presentation, placenta previa and known genetic disorders.

In all hospitals assisted births were carried out with the application of a vacuum instead of the use of a forceps.

In cases where indication to operative delivery was pathological cardiotocography, CTG traces were examined.

A board consisting of five among authors (AR, AS, PM, SD, AC) assessed appropriateness of indications for operative vaginal delivery, grouped as CTG patterns alterations, delay/arrest of second stage of labour or elective shortening of second stage of labour. Evaluation of CTG traces was blind to neonatal outcomes. In case of disagreement, a criterion of majority rule was applied.

Suitability criteria assessed by examination board on a Yes or No basis were:

- Suspected fetal compromise indicated by pathological CTG patterns. Unsuitability was defined also by objectively unreadable CTG traces;
- Delay or arrest of second stage of labour;
- Medical indication to avoid Valsalva maneuver (severe heart failure NYHA III and IV, untreated cerebrovascular malformations, disabling myopathies, history of retinal detachment and pneumothorax).

Maternal exhaustion or distress was not taken into consideration as an indication to vacuum procedure.

CTG traces were evaluated according to 2013 ACOG classification [9, 10]. They were fully analyzed in terms of baseline, variability, accelerations and decelerations. A CTG pattern was classified as “readable” when all the above-mentioned parameters could be objectively interpreted, when clinicians decided to perform a vacuum-assisted vaginal delivery.

In case of ACOG category 2–3 CTG patterns, vacuum application was considered appropriate by the board. On the contrary, it was considered inappropriate if performed with a category 1 CTG.

If a CTG trace couldn’t be analytically evaluated, vacuum application was considered inappropriate, due to unreadable CTG pattern.

We also evaluated transition phase duration (defined as the time between full cervical dilation and maternal urgency to push) and pushing stage. To the best of our knowledge, there is not a specified length of time above which the arrest of the second stage of labour can be diagnosed [11].

We adopted ACOG criteria, which define arrest of labour in the second stage if there is no descent or rotation of the presenting part after at least:

- three hours of pushing in nulliparous women
- two hours of pushing in multiparous women [12]

Pisa Hospital Committee for Medical and Health Research Ethics approved the study project (number of opinions: 14,061). Patients were thoroughly informed about the study upon admission and were informed about the possibility to be included in the study if operative vaginal delivery was performed. Women were given a specific factsheet.

Participant recruitment did not influence treatment strategies. Patients were managed according to usual clinical practice and to the judgment of the on-call obstetrician. Patients were free to withdraw consent at any time, without consequences on clinical care. If clinically indicated, vacuum was applied by the on-call obstetrician, prior verbal consent of the patient.

## Results

From April 2017 to January 2019, on a total of 9165 vaginal births, 468 women underwent vacuum-assisted delivery (5.1%) and were enrolled in our study.

466 patients met inclusion criteria; two women were excluded due to gestational age < 36 weeks. Patients’ characteristics are reported in Table 1.

**Table 1:** Patients’ characteristics

**Number of women**	466
Nulliparous (%)	382 (82)
Multiparous (%)	84 (18)
Median age (years) [IQR*]	32,08 [29 – 36]
Median gestational age (weeks) [IQR*]	40 [39 – 40]
**BMI < 30 kg/m**^**2**^** (%)** **BMI > 30 kg/m**^**2**^** (%)**	442 (94.9) 24 (5.1)
Induced labour (%)	149 (32)
Spotaneous labour (%)	317 (68)
**Previous cesarean section (%)**	38 (8.2)

^*^IQR interquartile range

The indications for vacuum assisted vaginal delivery were: ACOG category 2 CTG pattern in 242 cases (51.9%), ACOG category 3 CTG pattern in 11 cases (2.4%), medical indication to avoid Valsalva maneuver in 7 cases (1.5%), delay or arrest of second stage of labour in 123 cases (26.4%) and maternal exhaustion or distress in 83 cases (17.8%).

Data on transition from latent to active stage of labour, on duration of expulsive stage, use of epidural anesthesia and of oxytocin are reported in Table 2.

**Table 2:** Labour’s characteristics

Epidural analgesia (%)	229 (49,1)
Median transition phase duration [IQR*]	60 min [30 – 150]
Median pushing stage duration [IQR*]	67 min [35 – 120]
Oxytocin administration (%)	177 (38)

^*^IQR interquartile range

Operative Vaginal delivery failed in 17/466 women (3.6%), who subsequently underwent caesarean section. Forceps was never used in case of failure of vacuum delivery.

Neonatal and maternal outcomes are reported in Table 3. The only case of neonatal death was due to the failure of vacuum-application for arrest of fetal progression, with a subsequent cesarean section. The fetus was female-gendered and was born alive but seriously depressed, with a serious neonatal encephalopathy then followed by decease.

**Table 3:** Perinatal and maternal adverse events

Episiotomy (%)	181 (38.8)
Third and fourth degree lacerations (%)	19 (4.1)
Major postpartum hemorrhage (≥ 1500 ml) (%)	8 (1.7)
Number of blood transfusions	0
Maternal mortality	0
Maternal Intensive Care Unit admission	0
Newborns with an Apgar score at 5 min ≤ 7 and/or pH of the umbilical artery ≤ 7.00 (composite measure) (%)	1 (0.2)
HIE	0
Neonatal death (%)	1 (0.2)
Neonatal lesions (%)	1 (0.2)
Shoulder dystocia (%)	2 (0.4)

Among 242 vacuum-assisted deliveries due to ACOG category 2 CTG pattern, 72 (29.8%) were deemed appropriate, 63 (26%) were considered inappropriate while 107 (44.2%) resulted from unreadable CTG traces. Among 11 vacuum-assisted deliveries due to ACOG category 3 pattern, 1 (9.1%) was considered appropriate, 3 (27,3%) were deemed inappropriate, while 7 (63.6%) resulted from unreadable CTG traces.

Moreover, among the 7 vacuum-assisted deliveries performed to avoid Valsalva maneuver, 4 resulted to be inappropriate.

In addition, 62/123 (50.4%) vacuum-assisted deliveries due to delay or arrest of second stage of labour, resulted to be inappropriate.

In the examined population, we didn't record cases of pH < 7.00, Base excess > -12 or Apgar score < 7 at 5 min.

## Discussion

Our study described for the first time a heuristic approach to birth that we called “to get the baby out *off the hook*”. Obstetricians are constantly called upon to make decisions in labour ward. In order to do so, they have to identify significant data, make quick decisions and initiate appropriate care. In the best interest of the patient, medical decisions should be "rational", in order to maximize their chance of success. Therefore, decisions must be taken in accordance with the principles of logic, probability theory, rational choice theory, as well as with the best available evidence. Formal "medical decision analysis" and "evidence-based medicine" (EBM) meet these fundamental needs [12].

However, it is experimentally proven that even experts, instead of applying formal rules, often use simpler cognitive strategies, called heuristics. That is a method of facing issues that don’t follow a clear logical path, but rely on intuition and on circumstances of time [13]. Those strategies can succeed and prevent clinicians from complex reasoning and algorithm predictions. However, sometimes they mislead health professionals.

**Fig. 1:**
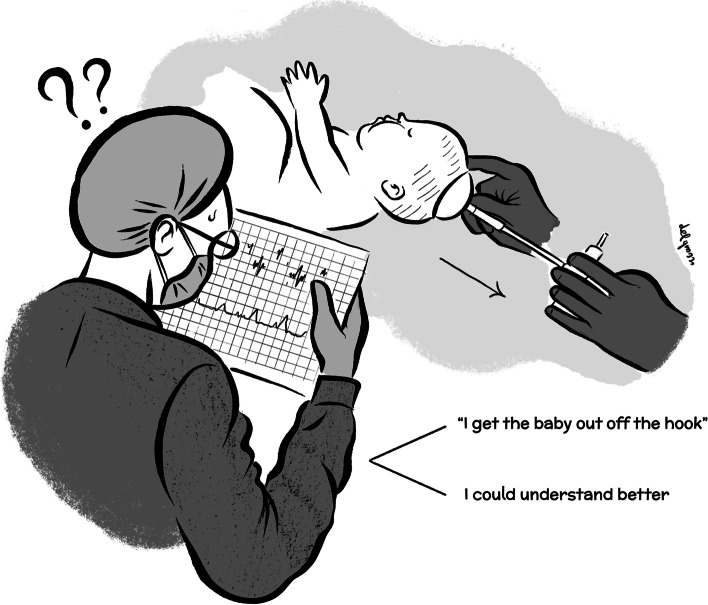
Clinicians in front of the choice

We found that operative vaginal delivery was performed in more than half of cases because of suspicious CTG alterations, but in half of these cases CTG interpretation was wrong or cardiotocographic traces were actually unreadable. In such uncertain situations, the majority of clinicians chose a prompt intervention and applied vacuum to speed delivery, while they should have shaped better decisions, placing a scalp electrode, evaluating maternal heart rate with pulse oximeter, performing ultrasound and/or changing maternal posture, interventions all available in the three Obstetric Units were our study was performed (Fig. 1).

In none of these three hospitals was the use of second line exams, such as STAN or fetal scalp blood sampling to determine pH, standardized. This can explain the high percentage of non-readable CTGs that were observed in our study.

Since a rapid assessment of pros and cons when time is running out is difficult, obstetricians in most cases preferred to act and apply vacuum, exposing mothers and babies to risks related to operative vaginal delivery, rather than trying to better understand clinical situation.

Human mind cognitive process uses numerous factors, at times subconscious, but not less systematic, to encode information and solve problems. Such factors deal with perception, construction of mental models and beliefs that influence the various *scenario* to be faced. They involve intrinsic reasons such as emotions and attitudes of the decision-maker. They are related also to memory, namely past experiences, which influence actual and future decisions. These elements are systematically affected by context and situations in which the decision is taken, causing relevant effects on clinical practice [14, 15].

In our study, doctors used a sort of heuristic that we named “to get the baby out *off the hook*” (Fig. 1). A heuristic is a mental shortcut that could help to manage a situation, without necessarily always guaranteeing a solution. Heuristics can be very effective, but at the same time they can lead to wrong decisions.

Although adverse effects due to operative vaginal delivery are not frequent, still there are some potential negative outcomes in terms of neonatal lesions, vaginal and perineal lacerations and traumatic experience reported by the patient [16, 17]. In our study, almost 1 in 20 women had third/fourth degree lacerations and neonatal complications also were reported, such as cephalohematoma and shoulder dystocia, including also one neonatal death.

Generally, women and babies are able to tolerate operative vaginal deliveries without running into serious complications. This causes birth operators to underestimate the possible side effects [18, 19]. This mechanism, which is really important in obstetrics and in every context with a low prevalence of adverse events, is known as “normalization of deviance” [20, 21].

The Hawthorne effect is probably present in the study, seeing as the gynaecologists have to fill in forms indicating the precise indication for the instrumental birth. We believe that this might have slightly brought to a reduction of inappropriate indications. Probably, for this reason inappropriate indications could be under-estimated. Another limit to our study is having expressed the appropriateness of such intervention solely on the main indication observed. In fact, the interpretation of the CTG traces and dystocia are not the only criteria for the decision to perform an OVD; characteristics of women, pregnancy and labor may also influence this complex choice.

Our study does not suggest a practical solution to difficult clinical choices taken in critical conditions but can make clinicians aware of this phenomenon.

Obstetricians should balance their decisions, using both emotional intelligence capabilities and rational process.

Within the method, the greater weight should be given to the self-criticism capacity, avoiding the general trend among professionals of self-absolution and self-justification [22].

## Conclusion

Our study shows for the first time that, having to choose whether to apply vacuum or not, more than half of obstetricians decided to adopt a heuristic approach in the absence of an appropriate diagnosis.

Using algorithms, made of a set of steps in order to solve specific problems, to better understand clinical situation, could instead help operators to process more information to reach clinical choice [23].

Most errors in clinical reasoning are not due to incompetence or inadequate knowledge of the subject, but to human vulnerability, under complex or uncertain conditions, and with little time available. Thus, errors related to decision-making process result from cognitive mechanisms and depend on how decisions are made [24, 25].

There is a need for practical medical training that take cognitive and heuristic process into account.

## Data Availability

The data that support the findings of this study are available from corresponding author A.S. but restrictions apply to the availability of these data, which were used under license for the current study, and so are not publicly available. Data are however available from the authors upon reasonable request and with permission of corresponding author A.S.
